# Adherence to oral zinc supplementation in the management of acute diarrhoeal disease among under-5 children: A systematic review and meta-analysis

**DOI:** 10.1017/S0950268825100733

**Published:** 2025-11-03

**Authors:** Somen Kumar Pradhan, Swagatika Pati, Pritimayee Sethy, Harshita Rajesh Dhusiya, Ashutosh Panda, Debasish Pandit, Jaya Singh Kshatri, Srikanta Kanungo, Sanghamitra Pati

**Affiliations:** 1 Community Medicine, Maharaja Krishna Chandra Gajapati Medical College and Hospital, Brahmapur, India; 2 Regional Medical Research Centre Bhubaneswar, Bhubaneswar, India; 3 Community Medicine, Shri Jagannath Medical College and Hospital, Puri, India; 4 Maastricht University, Maastricht, Netherlands; 5 Department of Health Research, Indian Council of Medical Research, New Delhi, India

**Keywords:** diarrhoea, medication adherence, zinc, under-5, compliance

## Abstract

Zinc supplementation is a critical adjunct therapy for managing acute childhood diarrhoea, particularly in low-income countries (LICs) and lower middle-income countries (LMICs). However, adherence to the recommended zinc regimen remains a major challenge, limiting its effectiveness in real-world settings. This systematic review and meta-analysis aimed to estimate the pooled adherence rates to zinc supplementation for diarrhoea in children under 5 and identify key determinants of adherence. A comprehensive search of PubMed, Embase, Scopus, Google Scholar, ProQuest, and CINAHL was conducted between 2000 and 2024. A total of 10 observational studies were included, with pooled adherence of 63.45% (95% CI: 51.62–75.28) for 10 days regimen and 34.58% (95% CI: 7.08–62.09) for 14 days regimen, along with high heterogeneity. Sensitivity analysis confirmed robustness of these estimates. Key factors associated with adherence included caregiver education, provider counselling, medication acceptability, and economic constraints related to caregiver buying capacity. Doi plot asymmetry suggested possible publication bias for 10 and 14 days regimen. Overall, adherence to zinc therapy remains sub-optimal, particularly for 14 days regimen compared to 10 days regimen. Targeted interventions addressing behavioural, provider, and formulation related barriers are urgently needed to optimize zinc adherence and improve diarrhoea outcomes globally.

## Introduction

Acute diarrhoeal diseases continue to pose significant public health challenges globally, particularly among children under 5 years of age. Despite advancements in health care, acute diarrhoea remains a leading cause of morbidity and mortality in this age group, accounting for approximately 444000 deaths annually, with a disproportionate burden in lower middle-income countries (LMICs) [1, 2]. In addition to mortality, acute diarrhoeal diseases contribute to substantial morbidity, including the triple burden of diarrhoea, stunting, and chronic diseases, while significantly affecting cognitive development [3–5]. In recent years, acute diarrhoeal diseases have also been a major contributor to the global burden of disease, causing a significant loss of disability-adjusted life-years (DALYs). In 2021, acute diarrhoeal diseases accounted for over 57.38 million DALYs globally, with LMICs accounting for the largest proportion [6–9].

Zinc supplementation has emerged as a cornerstone intervention for managing acute diarrhoea following evidence of its role in reducing diarrhoeal duration, severity, and recurrence [10]. In 2004, the World Health Organization (WHO) and United Nations International Children’s Emergency Fund (UNICEF) included zinc supplementation, alongside oral rehydration salts (ORS), in the standard treatment protocol for acute diarrhoea in children [11]. This decision was based on robust clinical evidence demonstrating that oral zinc supplementation for 10–14 days significantly improved outcomes compared to ORS alone [12–14]. In addition, as per a Cochrane review, the combined use of ORS and zinc has been shown to reduce the risk of persistent diarrhoea by 27% [15].

Zinc exerts its therapeutic effects through multiple biological mechanisms like enhancing mucosal integrity, modulating epithelial barrier function, boosting immune response, and restoring electrolyte balance, thus complementing the rehydrating effects of ORS [16, 17]. Nonadherence to zinc supplementation compromises these benefits, leading to prolonged episodes, increased recurrence, and heightened risk of complications, which, in turn, escalate morbidity, mortality, and healthcare costs associated with acute diarrhoea [18–20]. Despite clear guidelines and the proven efficacy of zinc supplementation, ensuring adherence to the full course remains a significant challenge, particularly in lower middle-income countries (LMICs). Key barriers include caregiver misconceptions about zinc’s role, inadequate awareness of its benefits, and poor taste acceptability, compounded by adverse effects like regurgitation and vomiting [21, 22]. Logistical challenges such as inconsistent supply chains, lack of trained healthcare providers, and affordability issues further hinder adherence in many low-resource settings [23–25]. These barriers, supported by moderate-certainty evidence, lead to sub-optimal adherence to national and global diarrhoea control programmes and threaten the effectiveness of zinc as a part of integrated diarrhoea management strategies.

Establishing pooled adherence rates through meta-analysis is imperative to understand the magnitude of the problem, identify trends, and recognize the determinants of poor adherence to zinc across diverse settings. This systematic review aimed to bridge knowledge gaps by quantifying zinc adherence rates, identifying barriers to compliance, and offering recommendations to improve adherence among under-5 diarrhoea cases. The results will be instrumental in guiding programme implementers and policymakers in scaling up effective interventions for diarrhoea management, ultimately improving child health outcomes and reducing diarrhoea-related DALY loss.

## Materials and methods

### Search strategy

We conducted a comprehensive literature search across PubMed, Embase, Scopus, ProQuest, Google Scholar, and CINHAL databases to identify peer-reviewed articles relevant to our objective. Our focus was on observational studies assessing the compliance of under-5 children to prescribed regimen of zinc formulation in an event of acute diarrhoeal disease. The search encompassed publications in all languages, from 1 January 2000 up to 31 December 2024. While the screening process was primarily conducted in English, articles in other languages were reviewed with the aid of translation tools to ensure thorough coverage.

We developed a comprehensive search strategy using a combination of Medical Subject Headings (MeSH) terms and free-text keywords to identify studies assessing adherence to zinc supplementation in paediatric diarrhoea. The search was conducted in PubMed, ProQuest, Embase, CINAHL, and Scopus using controlled vocabulary and free-text terms related to zinc (e.g., ‘zinc’, ‘zinc supplementation’), adherence (e.g., ‘adherence’, ‘compliance’, ‘treatment adherence’), and diarrhoea (e.g., ‘diarrhea’, ‘diarrh*’, ‘loose motion’). The full search strategy, including database-specific adaptations, is detailed in Supplementary Appendix A. Additionally, reference lists of the included studies and related systematic reviews were examined to identify any further relevant articles. This systematic review followed the Preferred Reporting Items for Systematic Reviews and Meta-Analyses (PRISMA) guidelines, and the study protocol was pre-registered with OSF (doi: https://doi.org/10.17605/OSF.IO/R67FK).

### Selection criteria

Our systematic review includes studies that met the following criteria: (i) studies in which participants were children <5 years of age; (ii) zinc supplementation was used as a part of routine management of acute diarrhoeal disease; (iii) studies which reported the compliance/adherence to zinc regimen (10 days/14 days) as frequency or percentage; (iv) studies with an observational study design such as cross-sectional or cohort as well as prospective single-arm pragmatic or open-label interventional studies reporting real-world adherence. Studies with the following criteria were excluded: (i) zinc therapy as a part of trial to assess interventions to improve compliance; (ii) zinc used in treatment of any associated illness other than acute diarrhoeal disease; (iii) studies assessing the adherence of medical practitioners to usage of zinc in acute diarrhoeal disease; (iv) any other study designs such as randomized control trial, quasi-experimental designs, case–control studies and qualitative research; or (v) were opinion articles, conference presentations, books, letters, editorials, reviews, dissertations/theses, or abstracts.

### Data extraction

The process of screening and selection was done using Covidence software (Melbourne, Australia) [26]. Using the mentioned search strategy, the title and abstract of all the articles were screened by four independent reviewers (JSK, SP, PS, HRD) for their eligibility and relevance. This was followed by full-text screening of the eligible articles and any disagreements in the process were resolved using a fifth reviewer (SKP). Data were extracted using a standard format in Microsoft Excel Version 2021, to ensure consistency and completeness. Data were extracted under the domains which include study characteristics (e.g., author, publication year, study design and setting, sample size), population demographics (e.g., study location, socio-economic status, age group), and treatment and adherence particulars (e.g., type of zinc formulation, dose of zinc, duration of treatment, and percentage of compliance among the participants). Additionally, we also recorded the factors associated with compliance as reported in these studies.

### Quality assessment

We used the National Heart, Lung, and Blood Institute’s (NHLBI) Quality Assessment Tool for Observational Cohort and Cross-Sectional Studies to evaluate the quality of the studies (Supplementary Appendix B) [27]. The checklist assigns scores ranging from 0 to 6, which were used to categorize studies as follows: scores of 0 to 2 indicated a low risk of bias, 3 to 4 indicated some concern, and 5 to 6 indicated a high risk of bias.

### Statistical analysis

We performed the statistical analysis using MetaXL version 5.3 to determine the pooled compliance rate for zinc treatment in children [28]. Compliance rate was calculated by dividing the number of children adhering to the treatment (numerator) by the total sample size (denominator). In cases where the exact number of adherent children was not reported, the numerator was derived based on the provided compliance percentage. A pooled compliance rate, along with 95% confidence intervals (CIs), was computed for the overall population and relevant sub-groups. Given the expected heterogeneity in study designs, populations, and adherence measurement methods, a random-effects model (Der Simonian and Laird method) was employed to obtain more generalizable pooled estimates by accounting for both within-study and between-study variability [29]. Given that some studies reported adherence rates for both 10 and 14 days regimen, we conducted separate pooled analyses for these two treatment durations. The pooled prevalence of adherence for 10 and 14 days zinc supplementation was estimated independently, given the anticipated heterogeneity across studies. The individual study proportions, as well as the pooled effect estimates with 95% CIs, were visualized using forest plots. Heterogeneity among studies was assessed using the *I* ^2^ statistic, which measures the proportion of variability attributable to differences between studies rather than random chance. We also undertook sensitivity analysis to understand how individual studies influenced the magnitude of pooled adherence estimates in this meta-analysis. To evaluate potential publication bias, both Doi and funnel plots were utilized, providing a systematic approach to assess bias in the studies reporting zinc treatment compliance.

## Results

### Study selection and characteristics

**Figure 1.**
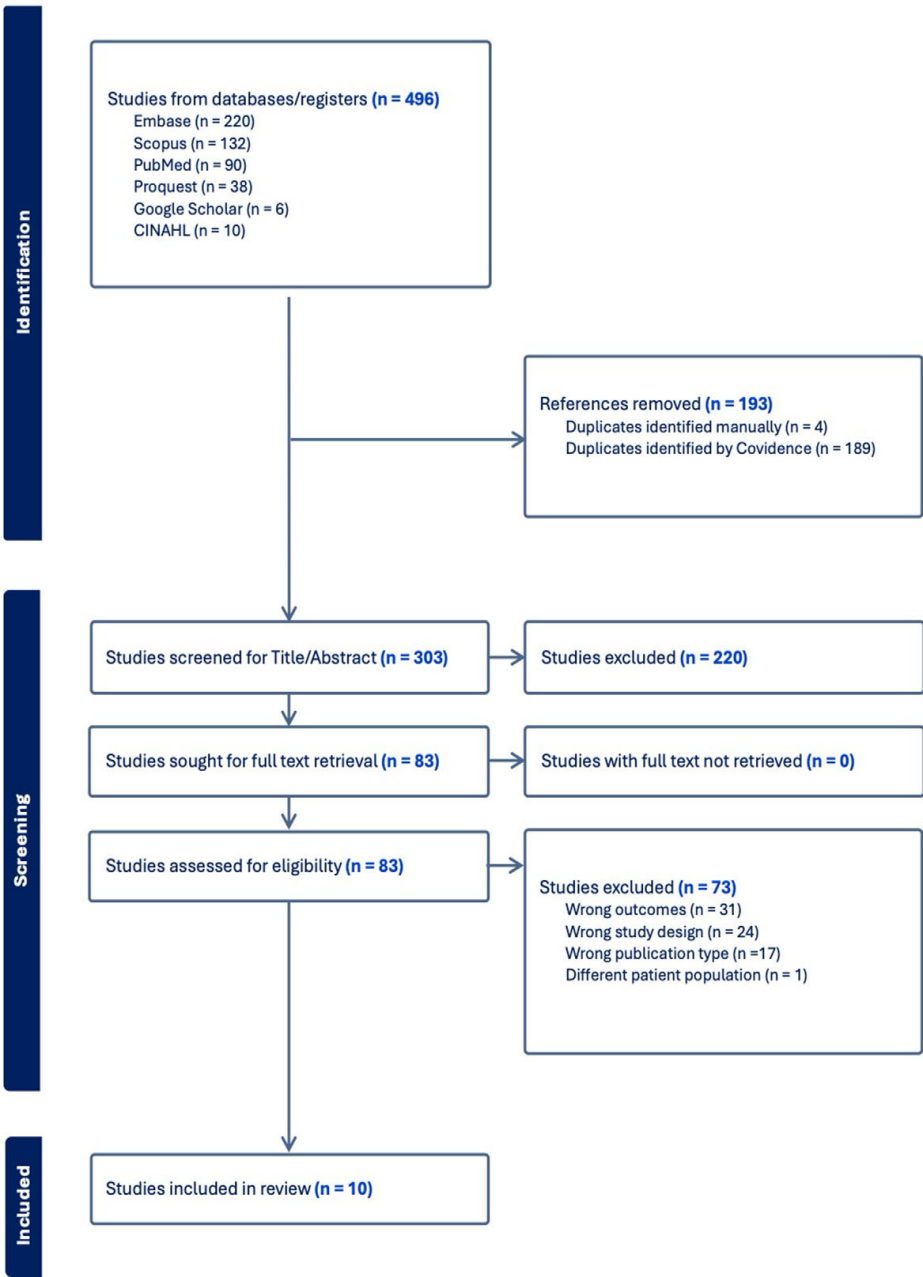
PRISMA flow diagram.

A total of 496 records were identified through database searches (Embase = 220, Scopus = 132, PubMed = 90, ProQuest = 38, Google Scholar = 6, CINAHL = 10). After removing 193 duplicates, 303 articles were screened, of which 220 were excluded based on title and abstract. The remaining 83 full-text articles were assessed for eligibility, leading to the exclusion of 73 studies due to wrong outcomes (*n* = 31), study design (*n* = 24), publication type (*n* = 17), and patient population (*n* = 1). Ultimately, 10 studies met the inclusion criteria and were included in the systematic review and meta-analysis (Figure 1).

Eight of the 10 included studies were conducted in lower middle-income countries (LMICs) (Bangladesh, India, Kenya, Pakistan, and Nigeria) and two from LICs (Ethiopia and Mali). Study periods ranged from 2004 to 2022, with sample sizes varying between 82 and 4180 participants. The study designs comprised cohort studies (*n* = 2) [30, 33], cross-sectional studies (*n* = 5) [31, 32, 34–36, 38, 39], and a prospective, single-arm, open-label interventional study [37], with settings distributed between hospital-based (*n* = 4) [32, 35, 37, 39] and community-based (*n* = 6) [30, 31, 33, 34, 36, 38] studies. Of the 10 included studies, six studies [30, 32–34, 37, 39] reported adherence data for 10 days zinc supplementation only, two studies [35, 38] reported adherence data for 14 days zinc supplementation only, and two studies [31, 36] provided adherence data for both 10 and 14 days regimen (Table 1). The definitions of diarrhoea and adherence used in the included studies have been detailed in Supplementary Appendix D.

**Table 1.** Adherence to zinc supplementation among under-5 diarrhoea children for various studies with study characteristics

Study ID	Country from where research participants were drawn	Type of country as per world bank classification	Study period	Study design	Study setting	Age group of study population	Doses of zinc prescribed	Zinc compound	Duration of zinc prescribed in days	Total sample size	Number of patients adherent completely	Adherence in percentage	Average duration of zinc received	Factors affecting Zn compliance
Nasrin et al. [30]	Bangladesh	LMIC	2004	Cohort	Community based	3–59 months	20 mg	NR	10	303	169	55.78%	7.78 days	A higher proportion of children who did not complete the full course was given other medicines apart from ORS and zinc (81% vs. 61%, *p* < 0.001).
Winch et al. [31]	Mali, West Africa	LIC	2004	Mixed method	Community based	0–60 months	10 mg (0–5 months); 20 mg (6–60 months)	NR	14	116	77	66.38%	NR	Community reaction to the zinc treatment was very favourable. Most parents reacted very positively to the effect of the zinc treatment. Parents particularly noted the increase in the child’s appetite and the return of normal activity levels (qualitative findings).
									10	116	108	93.10%		
Ogunrinde et al. [32]	Nigeria	LMIC	NR	Cross-sectional	Hospital based	1–59 months	NR	Zinc gluconate	10	4180	3159	75.57%	NR	The rate was higher in male (1444/1871; 77.2%) than in female caregivers (1715/2309; 74.3%). Those who had no formal education adhered better than literate caregivers (χ^2^ = 140.6; *p* < 0.001).
Ahmed et al. [33]	Bangladesh	LMIC	2011	Cohort	Community based	6–59 months	20 mg	Zinc sulphate monohydrate	10	635	394	62.05%	NR	There was 63% excess risk for poor compliance to zinc if father stays at home with the family (OR = 1.63, 95% CI: 1.09–2.46, *p* = 0.019). Despite vomiting, 33% children continued to receive zinc tablet (OR = 0.67, 95% CI: 0.47–0.97, *p =* 0.032) after adjusting for covariates.
Simpson et al. [34]	Kenya	LMIC	NR	Cross-sectional	Community based	6–60 months	20 mg	NR	10	82	31	37.80%	6.8 days	Caregivers reported a very high level of satisfaction with zinc treatment, with 88% indicating that zinc (either in tablet or syrup form) was their most preferred treatment.
Valekar et al. [35]	India	LMIC	2014	Cross-sectional	Hospital-based	0–60 months	20 mg	NR	14	102	18	17.65%	NR	Mother’s education, father’s occupation, and birth order of child were significantly associated factors for zinc adherence.
Lamberti et al. [36]	India	LMIC	2014	Cross-sectional	Community based	2–59 months	10 mg; 20 mg	Zinc sulphate (102), zinc acetate (8), zinc gluconate (3)	14	113	54	47.79%	10.7 days	Adherence to both dose and duration was lower among households below the poverty line (aOR = 0.45, 95% CI 0.22–0.92) and caregivers of children aged 7–59 months (aOR = 0.44, 95% CI: 0.22–0.87). Odds of adhering to the GoI/IAP guidelines on both dose and duration were higher among caregivers who received such advice from providers (aOR = 9.97, 95% CI: 4.10–24.25).
									10	113	72	63.7%		
Nuzhat et al. [37]	Bangladesh	LMIC	2022	A prospective, single-arm, open-label interventional study	Hospital-based	3–59 months	10 mg (0–6 months); 20 mg (7–60 months)	NR	10	302	250	82.8%		Zinc tablets were well tolerated by the majority of children (92.7%). Some experienced side effects such as vomiting (32.8%), regurgitation (19.5%), and nausea (7.4%). Nearly all caregivers (99.3%) expressed willingness to use the same formulation again in the future.
Khaliq et al. [38]	Pakistan	LMIC	2012–2018	Cross-sectional	Community based	0–59.9 months	NR	NR	14	4068	299	7.3%	NR	Intake of zinc was significantly higher in children over 1 year compared to children below 1 year.
Atnafu et al. [39]	Ethiopia	LIC	2019	Cross-sectional	Hospital-based	0–60 months	10 mg (0–6 months); 20 mg (7–60 months)	NR	10	405	140	35.45%	NR	Zinc adherence was significantly lower among caregivers without formal education (AOR = 0.34; 95% CI: 0.16–0.74), those without follow-up appointments (AOR = 0.40; 95% CI: 0.23–0.70), and when children experienced side effects (AOR = 0.35; 95% CI: 0.20–0.65). In contrast, adherence was higher among caregivers with good knowledge about zinc (AOR = 3.01; 95% CI: 1.73–5.24) and those who received information from both pharmacy dispensers and healthcare providers (AOR = 8.4; 95% CI: 4.66–15.13).

### Pooled adherence to zinc supplementation

**Figure 2.**
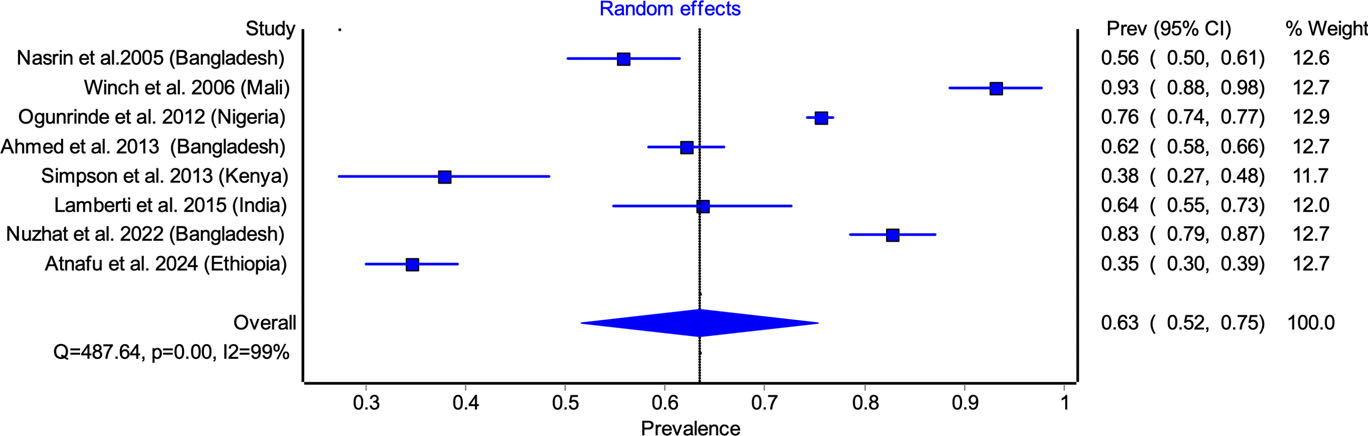
Pooled adherence to zinc supplementation for 10 days regimen.

**Figure 3.**
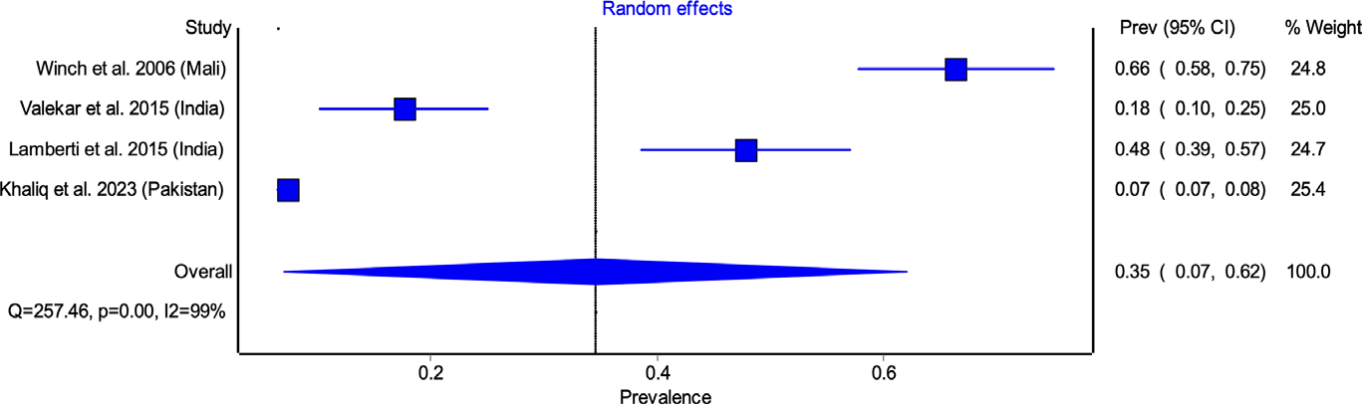
Pooled adherence to zinc supplementation for 14 days regimen.

Among studies reporting 10 days adherence, the pooled adherence rate was 63.45% (95% CI: 51.62–75.28) (Figure 2). The highest adherence rate was observed in Winch et al. [31] (93.10%), while the lowest was reported by Atnafu et al. [39] (34.57%). Significant heterogeneity was noted (*I* ^2^ = 98%, *p* < 0.05), likely attributable to differences in study settings, caregiver awareness, and healthcare access (Figure 2). Similarly, pooled adherence to the 14 days regimen was 34.58% (95% CI: 7.08–62.09) (Figure 3), with the highest adherence in Winch et al. [31] (66.4%) and the lowest in Khaliq et al. [38] (7.35%). (Figure 3).

### Sensitivity analysis

Sensitivity analysis was conducted by sequentially excluding each study to assess its impact on the pooled estimates. For 10 days adherence, exclusion of individual studies led to minor variations in the pooled prevalence estimates, ranging from 59.2% (95% CI: 46.6–71.7%), when Winch et al. [31] study was excluded, to 67.9% (95% CI: 58.7–77.2%), when Atnafu et al. [39] study was excluded (Supplementary Table S1). For 14 days adherence, the sensitivity analysis demonstrated a wider range of effects. The pooled prevalence was 23.9% (95% CI: 2.5–45.2%), when Winch et al. [31] was excluded, and 43.9% (95% CI: 14.5–73.2%), when Khaliq et al. [38] was excluded (Supplementary Table S2).

### Factors affecting zinc adherence

Multiple studies identified socio-demographic, healthcare-related, and behavioural determinants influencing adherence. Good caregiver knowledge about zinc substantially improves adherence (aOR = 3.01 in Ethiopia) [38], though the impact of education is complex and context specific, that is, better adherence was observed among caregivers with no formal education in Nigeria [32], whereas adherence was significantly lower among those without formal education in Ethiopia [39]. Economic status was another determinant, as households below the poverty line exhibited lower adherence rates [36]. High-quality counselling on dosing/duration significantly boosted adherence (aOR = 9.97 in India) [36], while receiving information from multiple sources (e.g., providers + pharmacists) had an exceptionally strong positive effect (aOR = 8.4 in Ethiopia) [39]. Additionally, child age (<1 year) and medication acceptability factors like taste, vomiting, and concurrent medication use was found to impact adherence negatively [30, 33].

### Publication bias

Publication bias was assessed using Doi plots and funnel plots. The Doi plot demonstrated major asymmetry for both the 10 and 14 days zinc adherence analyses (LFK index: −3.11 and 7.4, respectively), indicating substantial publication bias (Supplementary Figures S4 and S5). Funnel plot inspection of 10 and 14 days adherence analysis shows asymmetrical distribution of studies, indicating potential publication bias (Supplementary Figures S6 and S7). These findings highlight possible reporting discrepancies in studies assessing zinc supplementation and underscore the need for standardized reporting in adherence research.

### Quality and risk of bias assessment

Detailed results of the methodological quality and risk of bias assessment of individual studies according to the National Heart, Lung, and Blood Institute (NHLBI) Quality Assessment Tool are reported in Supplementary Appendix B. In summary, 6 (60%) studies were rated as having a low risk of bias, while 4 (40%) studies had some concerns. Common areas of bias included low participation rates (D3) and lack of sample size justification (D5), whereas all studies had clearly defined research objectives (D1) and study populations (D2). Despite these limitations, most of the studies maintained consistency in outcome assessment (D6), supporting their inclusion in the meta-analysis.

## Discussion

The findings from this systematic review and meta-analysis highlight critical insights into the adherence to oral zinc supplementation in the management of acute diarrhoeal disease among children under 5 years of age. While zinc has been established as an essential adjunct therapy to oral rehydration salts (ORS) in childhood diarrhoea, adherence remains sub-optimal, particularly in lower middle-income countries (LMICs) and low-income countries (LICs) [13]. This study identified a pooled adherence rate of 63.45% for the 10 days regimen and 34.58% for the 14 days regimen, emphasizing the challenges in sustaining zinc supplementation over extended periods. These findings have important implications for global child health policies and diarrhoeal disease management programmes [10].

Although earlier research dominated prior syntheses, this meta-analysis incorporates both historical and recent studies (up to 2024), providing a more comprehensive and updated understanding of zinc adherence. This addresses a critical gap in evidence synthesis, as the pooled estimates now reflect a broader time frame and offer valuable insights into adherence patterns and key influencing factors across diverse contexts. The findings can inform targeted interventions, guide policy adaptations, and emphasize the need for updated research to reflect contemporary healthcare challenges and improvements in zinc supplementation adherence.

The variation in adherence rates across different studies indicates that multiple determinants influence compliance with zinc supplementation. Key among these is the role of caregiver education and awareness. The influence of caregiver education on zinc adherence varied across settings, highlighting its context-specific nature. In our meta-analysis, there was a noticeable discrepancy in the relationship between caregiver education and zinc adherence, while some studies reported higher adherence among caregivers with limited formal education, others showed the opposite [22]. However, direct counselling from healthcare providers was strongly associated with improved adherence, supporting previous research indicating that adequate caregiver education and support are critical in ensuring treatment completion [40].

Economic status also emerged as a significant factor influencing adherence. Households below the poverty line had significantly lower adherence rates, likely due to financial constraints in purchasing zinc supplements when not freely available through public health programmes. This underscores the need for continued government and non-governmental support in ensuring free or subsidized zinc access, particularly in resource-limited settings [7, 19].

Medication acceptability played another crucial role in adherence, as adverse effects such as vomiting and regurgitation were commonly cited reasons for non-compliance. Studies also revealed that children who experienced vomiting were less likely to complete the prescribed regimen [41]. This finding suggests that alternative formulations or strategies to minimize side effects, such as improved palatability or dispersible tablets, could enhance adherence rates [42, 43].

The notable difference in adherence between the 10 (63.45%) and 14 days (34.58%) regimen underscore the challenges associated with extended supplementation periods. The declining adherence over 2 weeks suggests that a shorter course of treatment may be more practical in real-world settings. This aligns with previous studies indicating that shorter treatment durations or lower dose generally have higher compliance rates with similar effectiveness due to reduced caregiver fatigue and fewer chances of treatment discontinuation [44, 45]. This is particularly relevant for large-scale public health programmes, where ensuring high adherence over longer durations can be logistically challenging. Evidence from programmatic efforts from Gujarat and Uttar Pradesh in India demonstrated that targeted distribution of zinc and ORS through government channels can significantly improve access and adherence among the poorest populations [46, 47]. Moreover, the current WHO recommendation of a 10- and 14-day course introduces ambiguity in national policy adaptation, as it allows variation in programmatic implementation without clear evidence on the optimal duration. This lack of specificity may result in inconsistent guidance to caregivers and health workers, which in turn can affect adherence.

The observed heterogeneity in adherence rates across studies highlights the critical role of healthcare system in ensuring zinc supplementation compliance. In settings where healthcare providers actively promoted zinc alongside ORS, adherence rates were significantly higher [48, 49]. This finding is consistent with previous research emphasizing the importance of integrating zinc into routine diarrhoea management protocols at all levels of care. Furthermore, strengthening community health worker involvement and ensuring adequate stock of zinc supplements in healthcare facilities could mitigate some of the challenges identified in this study [9, 25]. The findings also suggest that incorporating zinc supplementation awareness into maternal and child health programmes could enhance compliance. Caregivers who received direct counselling from healthcare providers exhibited higher adherence, indicating that targeted educational interventions could bridge existing knowledge gaps. Mobile health (mHealth) interventions and behavioural change communication strategies could be leveraged to reinforce messages about the importance of completing the full zinc course [50, 51].

Publication bias analysis revealed major asymmetry for the 10 days adherence studies, indicating potential underreporting of negative findings or selective reporting in published literature [28]. This suggests the need for more comprehensive reporting standards in adherence research. Additionally, while the systematic review included a range of study designs, geographic regions, and time period, certain limitations must be acknowledged. First, significant heterogeneity was observed among included studies, likely due to differences in study settings, sample sizes, and methodologies. Second, the reliance on self-reported adherence measures in most studies could introduce reporting bias, as caregivers may overestimate compliance. Finally, the findings are largely derived from LICs and LMICs, limiting generalizability to high-income settings where different healthcare delivery models may influence adherence patterns [52, 53].

There is a pressing need for more comprehensive and standardized reporting in adherence research, including clear definitions and consistent outcome measurements. Future studies should explore innovative strategies to enhance adherence, including taste-masking formulations, alternative dosing regimens, and caregiver incentive programmes [4, 54]. Further research is also needed to assess the long-term impact of improved adherence on diarrhoeal outcomes, including recurrence rates and overall child morbidity and mortality. Our finding of higher adherence with the 10 days regimen should be interpreted cautiously, as clinical outcomes were not assessed, and future pragmatic trials are needed to link adherence with effectiveness.

## Conclusion

This systematic review and meta-analysis highlight the persisting challenges in achieving optimal adherence to oral zinc supplementation for childhood diarrhoea. While adherence to the 10 days regimen was relatively higher, the significantly lower compliance to the 14 days regimen suggests the need for programmatic shifts towards 10 days durations. Furthermore, the flexibility in the WHO’s 10–14 days recommendation may lead to varied interpretations across programmes. These findings are programmatically relevant, but further studies are needed to establish whether differences in adherence translate into clinical effectiveness. In the meantime, strengthening health education efforts, ensuring consistent zinc availability, and integrating adherence–promotion strategies into national diarrhoeal disease management policies remain critical to improving compliance rates and reducing diarrhoeal morbidity and mortality among under-5 children.

## Supporting information

10.1017/S0950268825100733.sm001 Pradhan et al. supplementary material Pradhan et al. supplementary material

## Data Availability

This systematic review is based entirely on data extracted from previously published studies. No new primary data were generated or collected, and as such, there are no additional data available for sharing. Registration**:** R67FK (Open Science Framework)

## Supplementary material

The supplementary material for this article can be found at http://doi.org/10.1017/S0950268825100733.
