# Supplementary material for: Adherence to oral zinc supplementation in the management of acute diarrhoeal disease among under-5 children: A systematic review and meta-analysis
Source: Epidemiol Infect. 2025 Nov 3;153:e129. doi: 10.1017/S0950268825100733 (PMC12641308; doi:10.1017/S0950268825100733)
Supplement: Pradhan et al. supplementary material [file S0950268825100733sup001.docx]

**Appendix A**

**Search strategy**

| **Database** | **No** | **Search Query** |  |
| --- | --- | --- | --- |
| **PubMed** | | | |
|  | #1 | "zinc"[Title/Abstract] OR "zinc"[MeSH Major Topic] |  |
|  | #2 | "Treatment Adherence and Compliance"[Mesh] OR "compliance"[Title/Abstract] OR "adherence"[Title/Abstract] |  |
|  | #3 | "diarrhea"[MeSH Major Topic] OR “diarrh*”[Title/Abstract] OR "loose motion"[Title/Abstract] |  |
|  | #4 | #1 AND 2# AND #3 AND |  |
| **ProQuest** | | | |
|  | #1 | TI,AB(zinc) OR MJMESH.EXACT(zinc) |  |
|  | #2 | MESH.EXACT("Treatment Adherence and Compliance") OR TI,AB(compliance) OR TI,AB(adherence) |  |
|  | #3 | MJMESH.EXACT(diarrhea) OR TI,AB(diarrh*) OR TI,AB("loose motion") |  |
|  | #4 | #1 AND #2 AND #3 |  |
| **Embase** | | | |
|  | #1 | zinc:ti,ab OR zinc/exp/mj |  |
|  | #2 | 'Treatment Adherence and Compliance'/exp OR compliance:ti,ab OR adherence:ti,ab |  |
|  | #3 | diarrhea/exp/mj OR diarrh*:ti,ab OR 'loose motion':ti,ab |  |
|  | #4 | #1 AND #2 AND #3 |  |
| **Cinhal** | | | |
|  | #1 | ( TI "zinc" OR MH "Zinc" ) |  |
|  | #2 | (MH "Treatment Adherence and Compliance+") OR (TI compliance OR AB compliance) OR (TI adherence OR AB adherence) |  |
|  | #3 | (MH "Diarrhea+") OR (TI diarrh* OR AB diarrh*) OR (TI "loose motion" OR AB "loose motion") |  |
|  | #4 | #1 AND #2 AND #3 |  |
| **SCOPUS** | | | |
|  | #1 | TITLE-ABS(zinc) OR INDEXTERMS(zinc) |  |
|  | #2 | INDEXTERMS("Treatment Adherence and Compliance") OR TITLE-ABS(compliance) OR TITLE-ABS(adherence) |  |
|  | #3 | INDEXTERMS(diarrhea) OR TITLE-ABS(diarrh*) OR TITLE-ABS("loose motion") |  |
|  | #4 | #1 AND #2 AND #3 |  |

**Table- S1: Sensitivity analysis of 10 days regimen of Zinc supplementation:**

| Excluded study | Pooled Prevalence | LCI 95% | HCI 95% | Cochran Q | p-value | I ^2^ | I ^2^ LCI 95% | I 2 HCI 95% |
| --- | --- | --- | --- | --- | --- | --- | --- | --- |
| Nasrin et al.2005 | 0.645413 | 0.516532 | 0.774293 | 452.5208 | 0.00000 | 98.67409 | 98.18035 | 99.03386326 |
| Winch et al. 2006 | 0.591539 | 0.465984 | 0.717094 | 405.465 | 0.00000 | 98.52022 | 97.94535 | 98.93424477 |
| Ogunrinde et al. 2012 | 0.61556 | 0.45201 | 0.779109 | 413.9813 | 0.00000 | 98.55066 | 97.99211 | 98.95383337 |
| Ahmed et al. 2013 | 0.636019 | 0.496893 | 0.775145 | 456.3763 | 0.00000 | 98.6853 | 98.19733 | 99.04117508 |
| Simpson et al. 2013 | 0.668605 | 0.546228 | 0.790982 | 445.5342 | 0.00000 | 98.6533 | 98.1488 | 99.02031286 |
| Lamberti et al. 2015 | 0.634033 | 0.50554 | 0.762525 | 483.9235 | 0.00000 | 98.76013 | 98.31023 | 99.09024971 |
| Nuzhat et al. 2022 | 0.606028 | 0.469938 | 0.742119 | 463.0816 | 0.00000 | 98.70433 | 98.22613 | 99.05362138 |
| Atnafu et al. 2024 | 0.679492 | 0.586946 | 0.772038 | 217.1922 | 0.00000 | 97.23747 | 95.87308 | 98.1507813 |

**Table-S2: Sensitivity analysis of 14 days regimen of Zinc supplementation:**

| Excluded study | Pooled Prevalence | LCI 95% | HCI 95% | Cochran Q | p-value | I ^2^ | I ^2^ LCI 95% | I ^2^ HCI 95% |
| --- | --- | --- | --- | --- | --- | --- | --- | --- |
| Winch et al. 2006 | 0.23866355 | 0.025008788 | 0.45231831 | 80.4262651 | 0.00000 | 97.51325 | 95.18293 | 98.7162467 |
| Valekar et al. 2015 | 0.40338599 | -0.00625142 | 0.81302339 | 251.216221 | 0.00000 | 99.20387 | 98.74139 | 99.49641289 |
| Lamberti et al. 2015 | 0.30241865 | -0.01293182 | 0.61776913 | 186.198725 | 0.00000 | 98.92588 | 98.21865 | 99.35232334 |
| Khaliq et al. 2023 | 0.43867347 | 0.144965443 | 0.73238149 | 73.8415121 | 0.00000 | 97.2915 | 94.66032 | 98.62613528 |

**Figure 4: Funnel plot for publication bias for 10 days regimen:**

**Figure 5: DOI plot for publication bias for 10 days regimen:**

**Figure 6: Funnel plot for publication bias for 14 days regimen:**

**Figure 7: DOI plot for publication bias for 14 days regimen:**

**Appendix B**

**Risk of bias assessment**


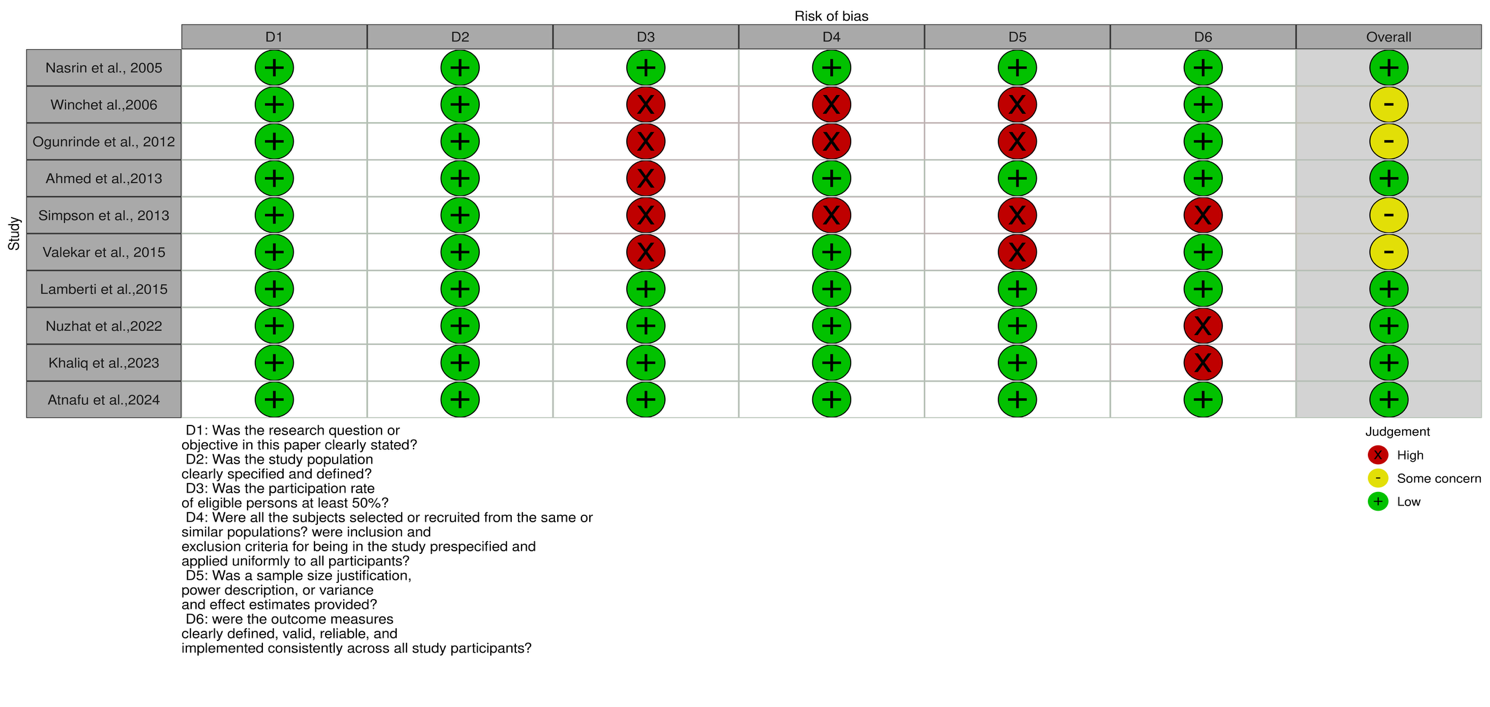


**Appendix C**

**PRISMA Checklist**

| **Section/topic** | **#** | **Checklist item** | **Reported on page #** | |
| --- | --- | --- | --- | --- |
| **TITLE** | | |  | |
| Title | 1 | Identify the report as a systematic review, meta-analysis, or both. | Pg. 1 | |
| **ABSTRACT** | | |  | |
| Structured summary | 2 | Provide a structured summary including, as applicable: background; objectives; data sources; study eligibility criteria, participants, and interventions; study appraisal and synthesis methods; results; limitations; conclusions and implications of key findings; systematic review registration number. | Abstract (Pg. 3) | |
| **INTRODUCTION** | | |  | |
| Rationale | 3 | Describe the rationale for the review in the context of what is already known. | Introduction (Pg. 5) | |
| Objectives | 4 | Provide an explicit statement of questions being addressed with reference to participants, interventions, comparisons, outcomes, and study design (PICOS). | Methods (Pg. 6) | |
| **METHODS** | | |  | |
| Protocol and registration | 5 | Indicate if a review protocol exists, if and where it can be accessed (e.g., Web address), and, if available, provide registration information including registration number. | Methods (Pg.6) | |
| Eligibility criteria | 6 | Specify study characteristics (e.g., PICOS, length of follow-up) and report characteristics (e.g., years considered, language, publication status) used as criteria for eligibility, giving rationale. | Methods (Pg.5) | |
| Information sources | 7 | Describe all information sources (e.g., databases with dates of coverage, contact with study authors to identify additional studies) in the search and date last searched. | Methods (Pg.6) | |
| Search | 8 | Present full electronic search strategy for at least one database, including any limits used, such that it could be repeated. | Methods(Pg.5)  Appendix A | |
| Study selection | 9 | State the process for selecting studies (i.e., screening, eligibility, included in systematic review, and, if applicable, included in the meta-analysis). | Methods (Pg.6) | |
| Data collection process | 10 | Describe method of data extraction from reports (e.g., piloted forms, independently, in duplicate) and any processes for obtaining and confirming data from investigators. | Methods (Pg.6) | |
| Data items | 11 | List and define all variables for which data were sought (e.g., PICOS, funding sources) and any assumptions and simplifications made. | Methods (Pg. 7) | |
| Risk of bias in individual studies | 12 | Describe methods used for assessing risk of bias of individual studies (including specification of whether this was done at the study or outcome level), and how this information is to be used in any data synthesis. | Methods (Pg. 7) | |
| Summary measures | 13 | State the principal summary measures (e.g., risk ratio, difference in means). | Methods (Pg.7) | |
| Synthesis of results | 14 | Describe the methods of handling data and combining results of studies, if done, including measures of consistency (e.g., I^2^) for each meta-analysis. | Methods (Pg.7) | |
| Risk of bias across studies | 15 | Specify any assessment of risk of bias that may affect the cumulative evidence (e.g., publication bias, selective reporting within studies). | Methods (Pg.8) | |
| Additional analyses | 16 | Describe methods of additional analyses (e.g., sensitivity or subgroup analyses, meta-regression), if done, indicating which were pre-specified. | Methods (Pg.7) | |
| **RESULTS** | | |  |  |
| Study selection | 17 | Give numbers of studies screened, assessed for eligibility, and included in the review, with reasons for exclusions at each stage, ideally with a flow diagram. | Figure 1 |  |
| Study characteristics | 18 | For each study, present characteristics for which data were extracted (e.g., study size, PICOS, follow-up period) and provide the citations. | Table 1 |  |
| Risk of bias within studies | 19 | Present data on risk of bias of each study and, if available, any outcome level assessment (see item 12). | Appendix B |  |
| Results of individual studies | 20 | For all outcomes considered (benefits or harms), present, for each study: (a) simple summary data for each intervention group (b) effect estimates and confidence intervals, ideally with a forest plot. | Table 1 |  |
| Synthesis of results | 21 | Present results of each meta-analysis done, including confidence intervals and measures of consistency. | Figure 2,  Figure 3 |  |
| Risk of bias across studies | 22 | Present results of any assessment of risk of bias across studies (see Item 15). | Appendix B |  |
| Additional analysis | 23 | Give results of additional analyses, if done (e.g., sensitivity or subgroup analyses, meta-regression [see Item 16]). | Table S1, Table S2 |  |
| **DISCUSSION** | | |  |  |
| Summary of evidence | 24 | Summarize the main findings including the strength of evidence for each main outcome; consider their relevance to key groups (e.g., healthcare providers, users, and policy makers). | Discussion (Pg. 15) |  |
| Limitations | 25 | Discuss limitations at study and outcome level (e.g., risk of bias), and at review-level (e.g., incomplete retrieval of identified research, reporting bias). | Discussion (Pg.16) |  |
| Conclusions | 26 | Provide a general interpretation of the results in the context of other evidence, and implications for future research. | Conclusion (Pg.16) |  |
| **FUNDING** | | |  |  |
| Funding | 27 | Describe sources of funding for the systematic review and other support (e.g., supply of data); role of funders for the systematic review. | NA |  |

**Appendix D**

**Definitions of Diarrhoea and Zinc adherence Used in Included Studies**

| **Study ID** | **Definition of diarrhoea** | **Definition of Zinc adherence** | **Method of assessing adherence** |
| --- | --- | --- | --- |
| Nasrin et al. 2005 | Not reported | Adherence was defined in relation to the dose given, frequency of daily administration, duration of treatment, and preparation (dispersion) of the tablets. | Through interview of study participants (self-reported) |
| Winch et al. 2006 | Not reported | Not reported | By conducting follow-up interviews with all children prescribed zinc from health centres or CHWs in the home on days 3 and 14 after the consultation. |
| Ogunrinde et al. 2012 | Not reported | Not reported | Study-specific questionnaire was used to collect relevant data from each consenting care giver by trained resident community health workers. Caregivers’ practice was assessed by their administration of supplied 10-day supplementation of zinc gluconate dispersible tablets to their wards. |
| Ahmed et al. 2013 | Diarrhoea was defined as three or more loose, liquid, or watery stools or at least one loose stool containing blood in a 24-hour period. | Good compliance was defined as once-a-day intake of a 20 - mg zinc tablet that had been completely dissolved in a small amount of water in a teaspoon for 10 consecutive days. | Data was collected by a home-based follow-up visit within 2 - 3 weeks after beginning daily zinc administration. Information was collected at the health facility and household by trained research assistants who administered a field-tested questionnaire. |
| Simpson et al.2013 | Not reported | Not reported | A semi–structured oral survey with both open–ended and  close–ended questions was used to interview caregivers in their a homes. |
| Valekar et al.2015 | Not reported | Not reported | Telephonic calls were made on given telephone number on 3rd, 7th & 14th day zinc supplementation. |
| Lamberti et al. 2015 | Episode of diarrhoea (defined as ≥3 loose or watery stools in a  24–hour period) in the 7 days preceding the household visit | Proportion of caregivers that adhered to the provider–advised course of zinc therapy. | Caregiver was asked to retain the packaging from any treatments administered to the child during this period and was also shown how to use a pictorial tracking form to record days on which the child experienced diarrhoea and days on which the child was administered ORS and/or zinc syrup or tablets. |
| Nuzhat et al.2022 | Not reported | It was evaluated in terms of the dose given and the number of days the child took medicine with proper preparation (dispersion) of the tablets. | Participants were asked to visit  hospital on 11th day with empty blister pack and diary card. In  case of failure to visit hospital by caregiver, study health worker  visited the house on 12th day and collected information. |
| Khaliq et al.2023 | The disease is characterised by the passage of three or more stools of a loose consistency on any given day, which leads to fluid and electrolyte loss. | Not reported | This study was based on the secondary analysis of the Pakistan Demographic and Health Surveys (PDHS) where a standard questionnaire was used for caregivers to collect information on Zinc adherence. |
| Atnafu et al. 2024 | Not reported | If respondents provided 10 mg of zinc for 10 consecutive days for infants less than six months and/or 20 mg of zinc per day for 10 consecutive days for children 6 months to 5 years of age | Adherence of zinc and ORS supplementation was ascertained through mothers’/caregivers’ self-report and pill count. |
